# [Retracted] Toll‑like receptor 2 downregulates the cholesterol efflux by activating the nuclear factor‑κB pathway in macrophages and may be a potential therapeutic target for the prevention of atherosclerosis

**DOI:** 10.3892/etm.2024.12760

**Published:** 2024-11-04

**Authors:** Yongqiang Li, Shuxin Shen, Shoukun Ding, Lixia Wang

Exp Ther Med 15:198–204, 2018; DOI: 10.3892/etm.2017.5404

Following the publication of this paper, it was drawn to the Editor's attention by a concerned reader that certain of the oil red O staining data shown in Fig. 1A on p. 200 were strikingly similar to data appearing in different form in another article written by different authors at different research institutes that was already under consideration for publication at the journal *Experimental and Therapeutic Medicine*.

In view of the fact that the abovementioned data were already apparently under consideration for publication in another article at the time of this paper’s submission, the Editor of *Experimental and Therapeutic Medicine* has decided that this paper should be retracted from the Journal. The authors were asked for an explanation to account for these concerns, but the Editorial Office did not receive a reply. The Editor apologizes to the readership for any inconvenience caused.

